# A case of spinal nerve neurotoxicity with ropivacaine after combined spinal and epidural anesthesia

**DOI:** 10.1186/s40981-021-00476-2

**Published:** 2021-09-28

**Authors:** Tsukasa Shimauchi, Jun Yoshino, Naoyuki Fujimura

**Affiliations:** 1 Department of Anesthesiology, St. Mary’s Hospital, Kurume, Fukuoka, Japan; 2 Quebec Lung and Heart Institute, Laval University, 2725 Chemin Ste-Foy, Quebec, QC G1V 4G5 Canada; 3 Department of Anesthesiology and Critical Care Medicine, Graduate School of Medical Sciences, Kyushu University, Fukuoka, Japan

**Keywords:** Ropivacaine, Local anesthetic, Neurotoxicity, Electromyography

## Abstract

**Background:**

Neurotoxicity caused by a local anesthetic after regional anesthesia is a rare but serious problem for anesthesiologists. It is difficult to diagnose neurotoxicity from anesthetics because of the large number of possible diagnoses. In this case report, careful monitoring by neurological examinations helped to diagnose local neurotoxicity caused after epidural anesthesia.

**Case description:**

A 41-year-old pregnant woman who underwent emergency cesarean delivery under combined spinal-epidural anesthesia suffered left leg paralysis after surgery. Multiple neurological examinations (e.g., electromyography, nerve conduction study) revealed that the paralysis was induced by the neurotoxicity of ropivacaine. The neurological examinations were also useful to monitor the recovery process.

**Conclusions:**

This is the first clinical case report that describes the diagnosis of and recovery from local anesthesia-induced neurotoxicity monitored by electromyography and nerve conduction study. Neurological disorders caused by regional anesthetics should be carefully examined and diagnosed using these neurological examinations.

## Background

Neurotoxic injury associated with regional anesthesia is a rare but serious problem. The pathophysiology of spinal cord injury associated with regional anesthesia is divided into mechanical trauma, vascular injury, and neurotoxicity from local anesthetic [1]. Neurotoxicity is an anesthesia-related nerve injury that can occur as an isolated event or in conjunction with physical injury to the spinal cord or spinal nerve roots [2].

Local anesthetic-induced neurotoxicity is possibly associated with mitochondria dysfunction and activation of the apoptosis pathway [3]. Pathological conditions can be induced by local anesthetics, such as lidocaine and tetracaine, and to a lesser extent bupivacaine and ropivacaine [4]. Ropivacaine is considered to be a safe local anesthetic [5, 6]; however, we encountered one case of serious neurotoxic injury associated with epidural ropivacaine. Neurotoxicity from a local anesthetic is rare and hard to diagnose. Chemotherapy induced neurotoxicity is known to be monitored with neurological examinations [7, 8]; however, anesthesiologists are not familiar with the examinations used to examine neurological disorders. Here, we used electromyography (EMG), nerve conduction study (NCS), and needle EMG to identify the site of neurological injury. The examinations were useful to make a diagnosis and to follow the recovery of neurological function. This is the first case report of neurotoxicity from a local anesthetic followed by regular electrophysiological evaluations.

## Case presentation

We received written permission from the patient to publish this report. A 41-year-old pregnant woman (height 157 cm, weight 60 kg) was diagnosed with fetal distress and underwent emergency cesarean delivery. She was at 40 weeks of gestation and had no problems during pregnancy. A preoperative examination found a normal platelet count and coagulopathy. The platelet count was 1.62 × 10^5^/mm^3^, the international normalized ratio of prothrombin time was 1.03, and the activated partial thromboplastin time was 29.2 s. Anesthesia was induced with combined spinal-epidural anesthesia under left lateral decubitus position. Spinal anesthesia was performed using a 27-G Quincke spinal needle, and the first trial was successful. 2.4 mL of isobaric 0.5% bupivacaine was injected into the subarachnoid space at L4/5. The epidural catheter was placed at the L4/5 intervertebral space with a Tuohy needle. There were no immediate complications such as paresthesia or bleeding at this point. After spinal anesthesia, the patient had loss of sensation from Th6 to S5. Cesarean delivery was performed rapidly and the delivery went smoothly with an operation time of 45 min. Continuous epidural anesthesia was started at the end of the surgery at 4 mL/h. The epidural anesthesia was a mixture of ropivacaine (0.2%, 200 mL) and fentanyl (16 mL, 0.8 mg). Postoperative loss of sensation from Th6 to S5 did not change when going back to the recovery room. The next day, the dose of continuous epidural anesthesia was reduced to 2 mL/h, at which point we confirmed that she could move her legs. However, she claimed to feel surgical pain, and the dose was raised back up to 4 mL/h. After 3 h, she became unable to move the bilateral lower limbs. The continuous infusion of epidural anesthesia was suspended 25 h after surgery (post-operative day (POD) 1); yet, sensory and motor disturbance persisted in the left lower extremity into POD2. We removed the epidural catheter, but paralysis remained.

**Fig. 1:**
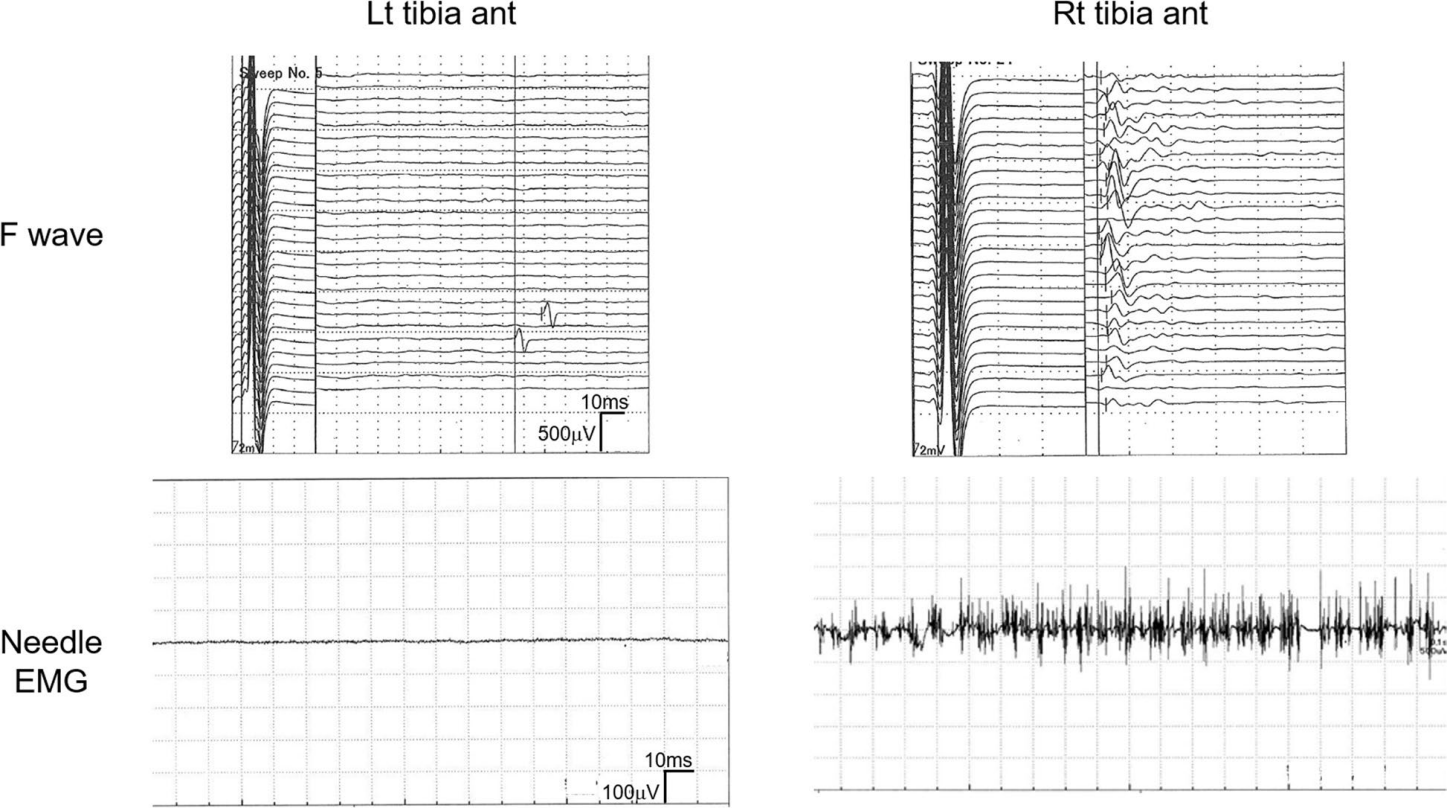
F wave frequency and needle EMG of the bilateral tibia anterior muscle recorded 7 days after the operation. F wave (upper) was recorded in 26 trials of supramaximal stimulation delivered at 1/s before a brief 20-Hz stimulation. There is no evoked F wave in the left tibial anterior muscle. Needle EMG (lower) shows no contraction and no action potential were recorded in the left tibial anterior muscle as well as impaired contraction in the right tibial anterior muscle. Lt tibia ant, left tibial anterior muscle; Rt tibia ant, right tibial anterior muscle

We consulted with a neurologist doctor to identify the cause of paralysis on POD2. Manual muscle test (MMT) performed in the lower limb muscle resulted with a score below 3 7 days post operation (Table 1). Tendon reflex in the left lower limb was absent while it was normal in the right lower limb. Babinski reflex, which is suggestive of pyramidal tract impairment, was negative in both legs. There were several possible diagnoses such as mechanical spinal injury, spinal cord infarction, brain infarction, and spinal cord hematoma. Spinal and brain magnetic resonance imaging revealed that there was no apparent cerebrospinal bleeding, infarction, or mechanical injury, but mechanical nerve injury could not be completely excluded. Neurological examination revealed lower left extremity listlessness and paresthesia, which deteriorated at the distal limb. EMG, which evaluates peripheral nerve injury as well as nerve root injury, showed impaired F wave appearance (Fig. 1). F wave is a response to antidromic activation of the motor neuron. NCS, which measures nerve conduction velocity in sensory and motor nerves, revealed that impairment of the sensory nerve conduction velocity (SCV) was more severe than that of the motor nerve conduction velocity (MCV). SCV in the left fibula, posterior tibia, and sural nerve were markedly lower. Needle EMG, which records spontaneous muscle contraction, showed the left tibia anterior muscle had no action potential, and left quadriceps and right tibia anterior muscles had decreased maximum interference patterns (Fig. 1).

**Table 1:** Lower limb muscle strength evaluated by manual muscle test. POD indicates post-operative days. POD1, POD7, POD54, POD106, and POD162. right limb/left limb. Ilio, iliopsoas muscle; Quad, quadriceps muscle; Ham, hamstring muscle; TA, tibialis anterior muscle; Gastro, gastrocnemius muscle; EHL, extensor hallucis longus muscle; FHL, flexor hallucis longus muscle; EDL, extensor digitorum longus muscle; FDL, flexor digitorum longus muscle

Muscle	POD2	POD7	POD54	POD106	POD162
Ilio	5/2	5/2	5/4	5/5	5/5
Quad	5/2	5/2	5/4	5/4	5/4
Ham	5/1	5/1	5/2	5/4	5/4
TA	5/1	5/1	5/2	5/3	5/4
Gastro	5/0	5/0	5/1	5/2	5/2
EHL	5/0	5/0	5/0	5/2	5/3
FHL	5/0	5//0	5/1	5/2	5/3
EDL	5/0	5/0	5/0	5/2	5/3
FDL	5/0	5/0	5/0	5/2	5/3

We considered possible diagnoses such as mechanical injury, epidural hematoma, spinal cord infarction, brain hemorrhage, delayed allergy, autoimmune disease, suppression by compression stocking, and neurotoxicity by bupivacaine. Mechanical nervous injury, epidural hematoma, and spinal cord infarction were excluded by spinal MRI. Brain hemorrhage and infarction were excluded by brain MRI plus computed tomography. Bilateral nerve impairment on EMG and the range of neurological manifestations negatively correlated with mechanical injury. Delayed-type hypersensitivity, known as type IV allergy, was excluded because of a negative result on a drug-induced lymphocyte test. Autoimmune diseases, such as Guillain-Barre syndrome and Miller Fisher syndrome, were excluded by the negative results from anti-GM1IgG and anti-GQ1bIgG antibody tests. Spinal cord neurotoxicity from bupivacaine, which was used for spinal anesthesia during surgery, was an important differential diagnosis in this patient. However, the confirmation of the movement of her lower limbs post-operation and the fact that the dose increase of continuous ropivacaine worsened the motor nerve disorder suggested there was no neurotoxicity from bupivacaine. Furthermore, absence of bladder rectal disorder and the range of neurological defect (L5 to L3, only left side) were also helpful in excluding neurotoxicity from bupivacaine.

**Fig. 2:**
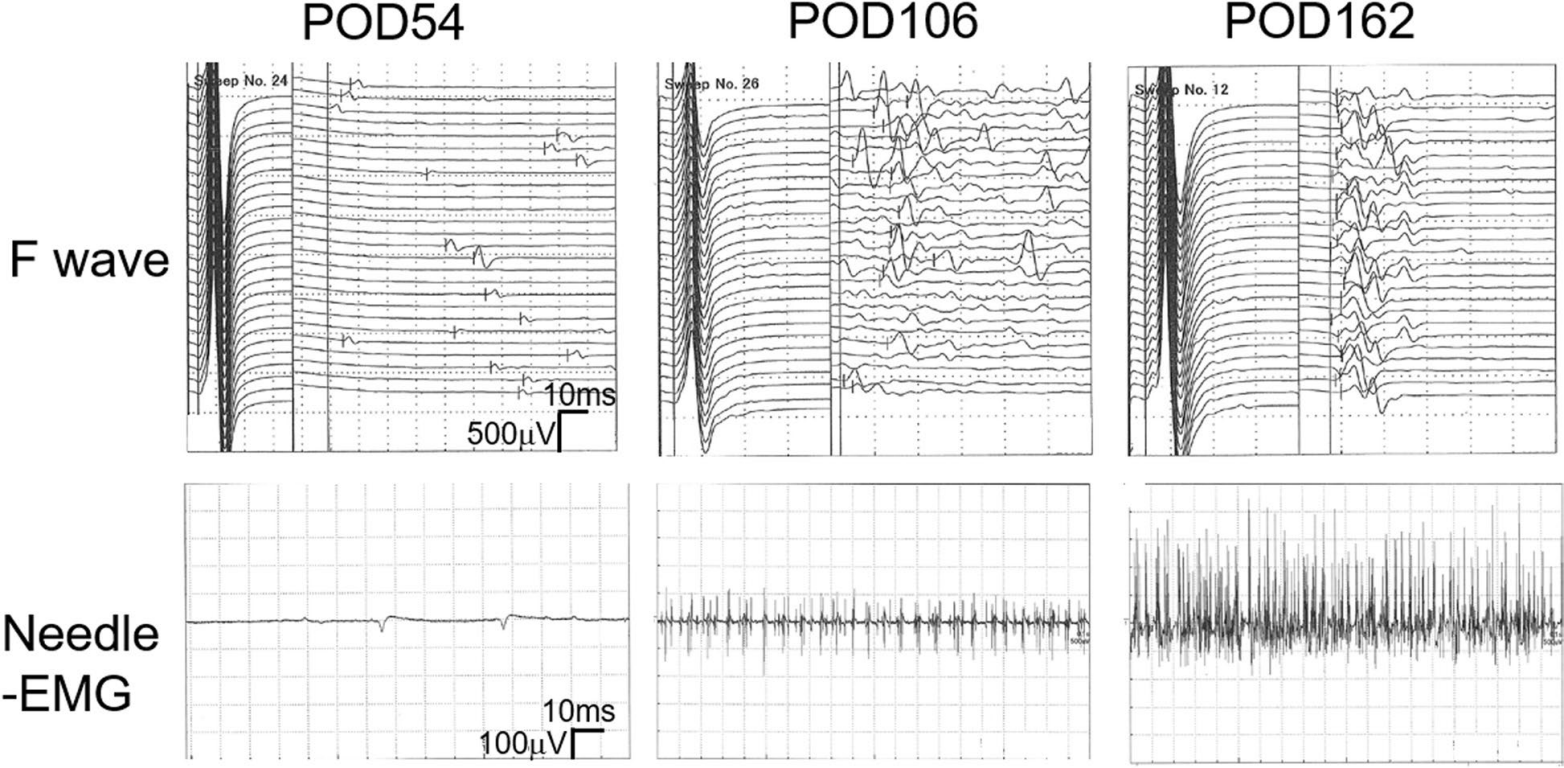
F wave frequency and needle EMG of the left tibia anterior muscle during the recovery process. POD indicates post-operative days. POD54 (left), POD106 (middle), and POD162 (right). This data clearly demonstrate that neurological disorder recovered gradually with time passes

After excluding the various possible diagnoses discussed above (mechanical injury, epidural hematoma, spinal cord infarction, brain hemorrhage, delayed allergy, autoimmune disease, mechanical suppression, and neurotoxicity by spinal anesthesia), the neurologist suggested a diagnosis of neurotoxicity from ropivacaine. The patient immediately began rehabilitation with physical therapies such as range of motion exercises, walking training, muscle strengthening exercises, and basic activity training. The neurological deficiency lasted for 50 days after surgery, after which small F waves appeared in the SCV simultaneously with clinical recovery (Fig. 2). MMT and basic activity recovered consistently with EMG recovery (Table 1). The EMG amplitude also increased over time. Tenacious rehabilitation was remarkably effective for the recovery of neurological disorder and she still has a little paresis but became able to walk by herself.

## Conclusion

This case report has two important findings for anesthesiologists. First, neurological detailed examination with EMG is useful to identify the site of neurological disorder after epidural anesthesia. Second, a neurological deficiency caused by neurotoxicity from local anesthesia does not readily recover, but continuous rehabilitation is beneficial for recovery.

In this case, EMG allowed the regional diagnosis of the neurological deficiency. A low SCV indicates myelin sheath damage and decreased EMG amplitude indicates axonal degeneration. The F wave, which is used to evaluate polyneuropathies [9], was also useful for assessing a neurological deficiency from ropivacaine-induced neurotoxicity. Needle EMG excluded a diagnosis of a bilateral neurological disorder from physical pressure caused by inadequate positioning. In addition, a pattern of degeneration in drug-induced neurotoxicity [10] known as “dying back” was observed. Impairment of sensory neurons was observed in the tibia peroneal and femoral nerves. Anesthesia-induced neurological deficiency usually appears on both legs; however, in this patient, neurological deficiency was much worse in the left leg than in the right. This abnormal event may be due to the placement of the epidural catheter more to the left side. These neurological observations and careful examination by EMG helped resolve the conclusive diagnosis. There are cases of neurological disorders after epidural anesthesia that are not diagnosed. This case emphasizes the importance of careful examination and monitoring by anesthesiologists for neurological deficiencies after epidural anesthesia. In addition, tenacious rehabilitation is important for recovery of neurological disorder with local anesthetics.

## Data Availability

Not applicable

## Abbreviations

EMG: Electromyography
MMT: Manual muscle test
MCV: Motor nerve conduction velocity
NCS: Nerve conduction study
POD: Post-operative day
SCV: Sensory nerve conduction velocity
